# Efficacy of Diclofenac and Indomethacin for Prevention of Post-Endoscopic Cholangiopancreatography (ERCP) Pancreatitis: A Systematic Review

**DOI:** 10.7759/cureus.66386

**Published:** 2024-08-07

**Authors:** Rudrani Kotha, Sabaa I Saad-Omer, Shivani Singh, Oluwatoba T Olayinka, Jaslin Orelus, Mah Rukh Nisar, Sondos T Nassar

**Affiliations:** 1 Internal Medicine, California Institute of Behavioral Neurosciences & Psychology, Fairfield, USA; 2 Neurology/Medicine, California Institute of Behavioral Neurosciences & Psychology, Fairfield, USA; 3 Medicine and Surgery, Jordan University of Science and Technology, Amman, JOR

**Keywords:** diclofenac sodium, indomethacin therapy, pharmacological prevention, pharmacology, : post ercp pancreatitis

## Abstract

Endoscopic cholangiopancreatography (ERCP) is a widely used diagnostic and therapeutic tool for pancreaticobiliary conditions. One of its major complications is pancreatitis. This study aims to understand the incidence of post-ERCP pancreatitis after using rectal diclofenac and Indomethacin as prophylactic measures. We retrieved 2870 articles from the PubMed, ScienceDirect, and Google Scholar databases. Using the Medical Subject Headings (MeSH) strategy in PubMed, we chose research articles published in the last five years. Exclusion criteria included paid full-text articles, abstracts, letters to editors, patients not undergoing ERCP, ages more than 45 years, animal studies, and non-English studies. The 2020 Preferred Reporting Items for Systematic Reviews and Meta-Analysis (PRISMA) criteria were used in the design of our systematic reviews. It was found that the medical world is still debating whether rectal diclofenac and Indomethacin are beneficial in avoiding post-ERCP pancreatitis (PEP). Rectal diclofenac is used. Although its effectiveness is debated due to mixed findings and concerns about certain outcomes, it is also considered beneficial in specific circumstances, such as before ERCP. Studies on rectal Indomethacin also yield contradictory results; while some emphasize the drug's large reduction in PEP incidence, especially in low-risk people, others question its efficacy. We need further studies to clarify the remaining uncertainties.

## Introduction and background

Pancreatitis, characterized by inflammation of the pancreas, poses a significant global health concern, with an annual occurrence estimated between 13 and 45 cases per 100,000 people. Its prevalence is on the rise, partly due to lifestyle choices like alcohol consumption, smoking, and obesity. While gallstones are the primary cause in developed nations, alcohol abuse is a prominent factor worldwide. Additional risk elements include genetic susceptibility, specific medications, infections, and metabolic conditions. Comprehending the epidemiology and risk factors is vital for devising effective preventive and therapeutic approaches such as endoscopic retrograde cholangiopancreatography [1,2].

Post-ERCP pancreatitis (PEP) stands as the most common and significant consequence of endoscopic retrograde cholangiopancreatography (ERCP), a widely employed therapeutic intervention for managing pancreaticobiliary diseases. However, this procedure carries notable risks. PEP impacts healthcare expenditures and patient well-being, affecting 1-9% of average-risk patients and 11-40% of high-risk patients [3,4]. Despite promising outcomes in reducing PEP incidence, endoscopists retain mixed sentiments regarding the use of preventive pancreatic duct stents (PDS) due to concerns over failure rates and associated consequences [5,6]. Furthermore, pharmacological prophylaxis, particularly rectal non-steroidal anti-inflammatory drugs (NSAIDs), has emerged as a viable preventive strategy, yet its efficacy across different risk categories remains uncertain [7,8]. Addressing the ongoing challenge of preventing PEP in ERCP procedures necessitates the exploration of practical preventive measures. Despite advancements, the prevalence of PEP continues to rise, highlighting the necessity for innovative therapies [9,10]. Pharmaceuticals, notably NSAIDs, have garnered interest due to their favorable risk profiles and ease of administration, potentially aiding in PEP reduction [11]. However, to establish their role as a standard preventive measure, further research is imperative to ascertain the efficacy of NSAIDs, particularly rectal administration of diclofenac or Indomethacin, in high-risk individuals [12].

A comprehensive study is indispensable in guiding healthcare practice, given the complexity and variability of preventive treatments and PEP risk factors. To address this gap, we aim to compare and evaluate the effectiveness of rectally administered NSAIDs, such as Indomethacin and Diclofenac, in reducing the incidence of PEP across various risk groups.

## Review

Methodology

(PRISMA) 2020 guidelines have been followed in this systematic review [13].

Eligibility criteria

Based on the participants’ intervention and outcome components, this review was created. Participants are individuals who are undergoing endoscopic retrograde cholangiopancreatography. Interventions include the use of rectal diclofenac or Indomethacin as a prophylaxis to prevent post-ERCP pancreatitis. Consequently, Table 1 below illustrates more of the inclusion and exclusion standards that were introduced.

**Table 1:** Details of inclusion and exclusion criteria RCT: Randomized controlled trial, ERCP: Endoscopic retrograde cholangiopancreatography

	Inclusion	Exclusion
Publication date	Last 5 years (2019-2024)	Above 5 years
Literature	Published literature	Author letters, conference abstracts, books, grey literature, non-published literature
Study types and designs	RCT, cohort, systematic reviews, meta-analysis, case reports, observational	
Population	Patients who are undergoing ERCP	Patients who are not undergoing ERCP
Sex	Both female and male	
Language	English	
Text availability	Free full text only	Abstracts paid full text, Letter to editors
Species	Humans	Animals
Age	Age less than 45	Age more than 45

Database and search strategy

A comprehensive search strategy was created to identify relevant studies in the following databases, including PubMed, Google Scholar, and ScienceDirect. The database was last searched in February 2024. Indomethacin, Diclofenac, ERCP, and pancreatitis were the terms entered into the search engines, and the Medical Subject Headings (MeSH) approach was employed in PubMed. Table 2 provides more details about PubMed and search techniques.

**Table 2:** Demonstrates more details about PubMed and search techniques

Databases	Keywords	Search strategy	Filters	Search result
PubMed	((((Indomethacin) OR (Diclofenac) AND (ERCP) AND (PANCREATITIS)	(Retrograde Cholangiopancreatography, Endoscopic)) OR (Cholangiopancreatographies, Endoscopic Retrograde)) OR (Endoscopic Retrograde Cholangiopancreatographies)) OR (Retrograde Cholangiopancreatographies, Endoscopic)) OR (Endoscopic Retrograde Cholangiopancreatography)) OR (ERCP)) OR ( "Cholangiopancreatography, Endoscopic Retrograde/adverse effects"[Majr] OR "Cholangiopancreatography, Endoscopic Retrograde/instrumentation"[Majr] OR "Cholangiopancreatography, Endoscopic Retrograde/methods"[Majr] OR "Cholangiopancreatography, Endoscopic Retrograde/mortality"[Majr] OR "Cholangiopancreatography, Endoscopic Retrograde/standards"[Majr] ) AND (Diclophenac)) OR (Dicrofenac)) OR (Dichlofenal)) OR (Diclofenac Sodium)) OR (Sodium Diclofenac)) OR (Diclofenac, Sodium)) OR (Diclofenac Potassium)) OR (Feloran)) OR (Voltarol)) OR (( "Diclofenac/administration and dosage"[Majr] OR "Diclofenac/adverse effects"[Majr] OR "Diclofenac/history"[Majr] OR "Diclofenac/immunology"[Majr] OR "Diclofenac/standards"[Majr] OR "Diclofenac/therapeutic use"[Majr] )) OR (Indometacin)) OR (Osmosin)) OR (Indocid)) OR (Metindol)) OR (Indomet 140)) OR (Indomethacin Hydrochloride)) OR (Amuno)) OR (Indocin)) OR (( "Indomethacin/administration and dosage"[Majr] OR "Indomethacin/adverse effects"[Majr] OR "Indomethacin/history"[Majr] OR "Indomethacin/immunology"[Majr] OR "Indomethacin/standards"[Majr] OR "Indomethacin/therapeutic use"[Majr] )) AND (Pancreatitis, Acute Edematous)) OR (Acute Edematous Pancreatitides)) OR (Edematous Pancreatitides, Acute)) OR (Edematous Pancreatitis, Acute)) OR (Pancreatitides, Acute Edematous)) OR (Acute Edematous Pancreatitis)) OR (Pancreatic Parenchymal Edema)) OR (Acute Pancreatitis)) OR (Acute Pancreatitides)) OR (Pancreatitis, Acute)) OR (( "Pancreatitis/congenital"[Majr] OR "Pancreatitis/epidemiology"[Majr] OR "Pancreatitis/etiology"[Majr] OR "Pancreatitis/genetics"[Majr] OR "Pancreatitis/history"[Majr] OR "Pancreatitis/immunology"[Majr] OR "Pancreatitis/pathology"[Majr] OR "Pancreatitis/physiopathology"[Majr] OR "Pancreatitis/prevention and control"[Majr] OR "Pancreatitis/therapy"[Majr] ))	Free full text, in the last 5 years, Humans, English, Middle-aged 45+ years	1,056 results
Google Scholar	ERCP AND Diclofenac OR Indomethacin AND Pancreatitis	"ERCP" AND "Diclofenac" OR "Indomethacin" AND "Pancreatitis"	2019-2024	1740 results
Science Direct	ERCP AND Diclofenac OR Indomethacin AND Pancreatitis	"ERCP" AND "Diclofenac" OR "Indomethacin" AND "Pancreatitis"	2019-2024 English Open access and open archive	74 results

All of the references were arranged sequentially in EndNote. We used Endnote and manual effort to eliminate the duplicates. Furthermore, the records underwent a title and abstract filter, enabling the removal of irrelevant publications. The entire text of the articles was obtained. The papers that were successfully retrieved were assessed using the appropriate quality assessment tool in order to reduce the possibility of bias in this investigation.

Quality assessment

The proper quality rating procedures were employed to assess the quality of the nominated studies. The systematic reviews and meta-analysis were assessed using the assessment of multiple systematic reviews (AMSTER) checklist [14]. The scale for the assessment of randomized controlled trials by the Cochrane assessment tool [15]. And finally, we used the Newcastle-Ottawa scale (NOS) to assess the cohort studies [16]. 

Data collection process

Following the data extraction process, every co-author made an equal contribution to the completeness of the received data. The primary outcomes, including the development of complications like pancreatitis following an ERCP procedure, were carefully scrutinized in each of the publications that were included in the shortlist. The majority of the results showed how the prophylactic action taken affected the outcome. 

Results

**Figure 1:**
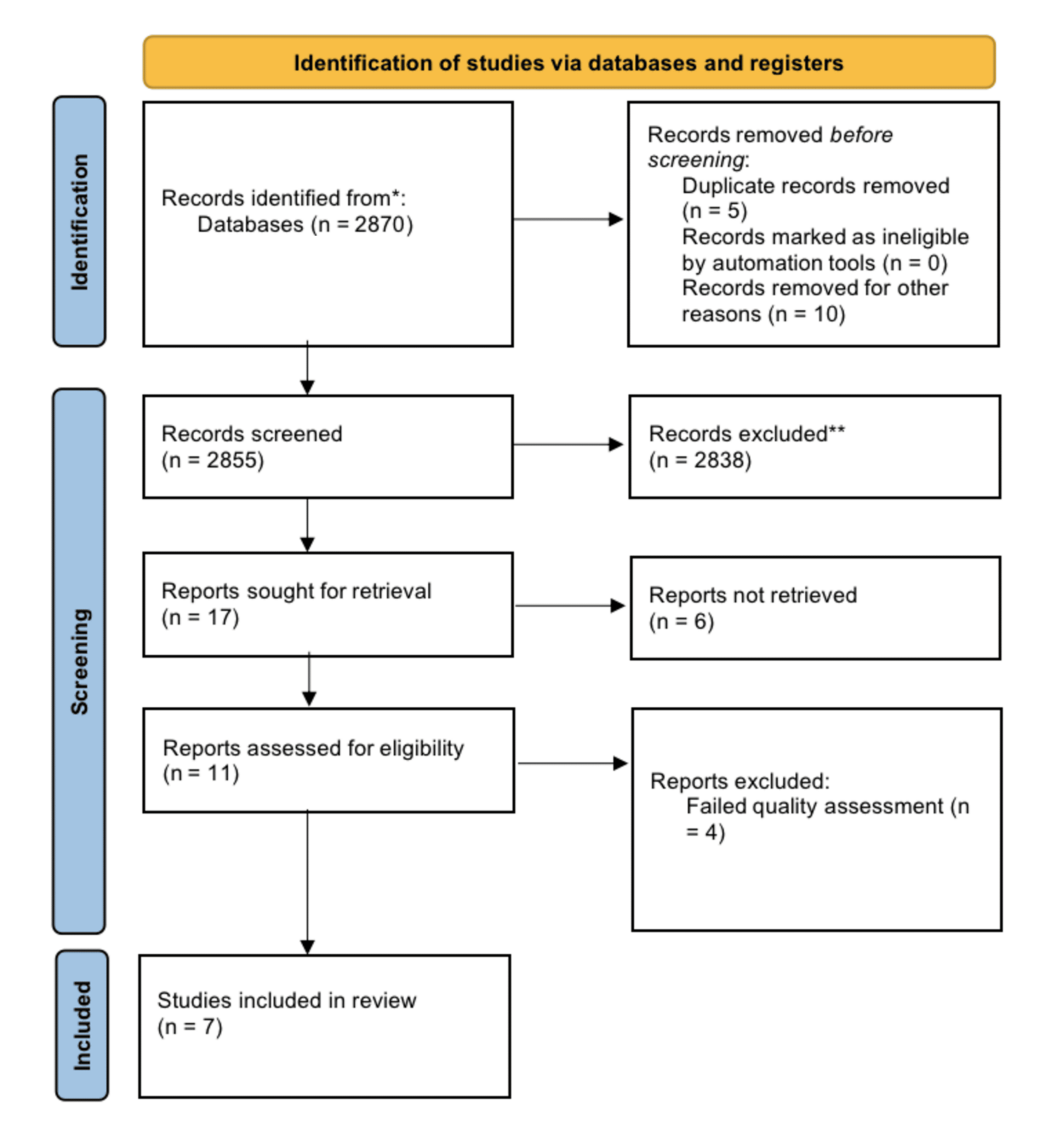
PRISMA 2020 flow diagram PRISMA: Preferred reporting items for systematic reviews and meta-analyses

In our first search of PubMed, Google Scholar, and Science Direct, we found 2870 articles. Five entries were removed as duplicates using EndNote. We manually screened the remaining 2855 articles using their titles, and 2838 of them were deleted. Seventeen articles were left for retrieval, while six were not. Seven articles remained based on the same eligibility criteria. Two distinct co-authors then used full-text, titles, abstracts, and extensive inclusion-exclusion criteria to screen the remaining seven papers. After a thorough screening process, we included publications that met our inclusion criteria, which included reviews of articles published in English within the last 10 years, observational studies, and non-randomized controlled trials, as well as any that were relevant to our research question. For a comprehensive quality/bias assessment utilizing standardized quality assessment methodologies, a total of 11 papers were included. After a quality analysis, four papers were found to be ineligible for this systematic review, leaving seven research studies to be included. Figure 1 shows the PRISMA 2020 flow diagram [13].

From the quality appraisal carried out, three cohort studies received an acceptable rating from the NOS assessment tool [16], as described in Table 3.

**Table 3:** Demonstrates the NOS assessment tool for the cohort studies in this review Results of the NOS assessment tool for observation studies by review authors: Passing score: 7/9, N/A: not applicable, NOS: Newcastle-Ottawa scale

	Representativeness of the exposed cohort	Selection of the non-exposed cohort	Ascertainment of exposure	Demonstration that the outcome of the interest was not present at the start of the study	Comparability of the cohort-based design /analysis	Assessment of the outcome	Was the follow-up long enough for the outcome to occur	Adequacy of follow-up cohorts	Pass/Fail
Mohamed et al. (2020)[17]	Yes	Yes	Yes	yes	Yes	Yes	Yes	Yes	Pass
Geraci et al., (2019) [18]	Yes	Yes	Yes	Yes	Yes	Yes	Yes	Yes	Pass
Hector et al. [19]	Yes	Yes	Yes	Yes	No	Yes	Yes	Yes	Pass

Two randomized controlled trial studies that passed the Cochrane assessment tool [15] were included in our analysis, as demonstrated in Table 4. 

**Table 4:** Demonstrates the Cochrane assessment tool for the randomized controlled trial studies in this review Results of the Cochrane assessment tool for randomized controlled trial studies by review author: N/S: Non-specified, LR: Low Risk, UNC: Unclear

Cochrane appraisal	Year of Study	Random sequence generation	Allocation concealment	Blinding of participants & personnel	Blinding of outcome assessment	Incomplete outcome data	Selective reporting	Risk of bias
Evan et al. [20]	2020	Yes	Yes	Yes	Yes	Yes	Yes	LR
Bimal et al. [21]	2021	Yes	N/S	No	N/S	N/S	N/S	UNC

Additionally, we used the AMSTER checklist [14] to evaluate the systematic review/meta-analysis which met the standards as described in Table 5. 

**Table 5:** Demonstrates the AMSTER checklist for systematic review and meta-analysis Results of the AMSTER checklist for systematic review/Meta-analysis by review authors: The passing score is >70%. AMSTAR: Assessment of multiple systematic reviews

First author, year	Was a prior design provided	Was there duplicate selection and data extraction	Was a comprehensive literature search performed	Was the status of publication (e.g., grey literature) used as an inclusion criteria	Was a list of studies (included and excluded provided)	Was the characteristic of the included studies provided	Was the scientific quality of the included study used appropriately in formulating a conclusion	Were the methods used to combine the findings of studies appropriate	Was the likelihood of publication bias assessed	Was the conflict of interest included
Juan et al. (2019) [22]	Yes	Yes	Yes	No	Yes	Yes	Yes	Yes	No	No
Shuang et al. (2021) [23]	Yes	Yes	Yes	No	No	Yes	Yes	Yes	Yes	Yes

Table 6 describes the articles retained in our review and their corresponding results. 

**Table 6:** Selected articles included in this review with results RCT: Randomized controlled trial; SR: Systemic review; MA: Meta analysis, ERCP: Endoscopic retrograde cholangiopancreatography

Authors	Study type	Results
Mohamed et al. (2020) [17]	Cohort	Rectal Indomethacin reduced the incidence and severity of post-ERCP pancreatitis
Geraci et al. (2019) [18]	RCT	The study showed that a single rectal administration of diclofenac, 30 to 90 minutes before ERCP, is an efficacious and safe measures for reducing the incidence and severity of post-ERCP pancreatitis
Hector et al. (2020) [19]	Cohort	Rectal diclofenac administered at the beginning of the ERCP did not reduce the PEP rate in this patient cohort.
Evan et al. (2020) [20]	RCT	Dose escalation to 200mg Indomethacin compared to standard 100mg Indomethacin does not confer any advantage.
Bimal et al. (2021) [21]	RCT	Prophylactic use of rectal Indomethacin or diclofenac during or just after ERCP significantly reduces the incidence of post-ERCP pancreatitis.
Juan et al. (2019) [22]	SR/MA	Rectal administration of diclofenac and indo- methane significantly reduced the risk of developing post-ERCP pancreatitis
Shuang et al. (2021) [23]	SR/MA	Rectal Diclofenac and Indomethacin do prevent the incidence of post-ERCP pancreatitis

Discussion

Assessing the Effectiveness of Rectal Diclofenac in Preventing Post-ERCP Pancreatitis

It has been suggested that phospholipase A2 plays a pivotal role in this inflammatory response, regulating proinflammatory mediators like prostaglandins, leukotrienes, and platelet-activating factors. It is widely accepted that the physiopathological event that triggers PEP is the local and systemic inflammatory response induced by ERCP; in vitro assays demonstrate that non-steroidal anti-inflammatory drugs (NSAIDs) like diclofenac are potent inhibitors of phospholipase A2 [18].

According to the research done by Hector and others, rectal diclofenac is inexpensive and has a good safety record, but it's uncertain how effectively it works to prevent post-ERCP pancreatitis (PEP). The gender distribution in the evaluated biodemographic characteristics revealed a female predominance in both groups, with no significant variations between them. (68% vs. 69%) (p = 0.933). Groups A and B had similar average ages of 61.9 ± 17.8 and 58.3 ± 15.8 years, respectively (p = 0.2606). Prophylactic encyclopedic medication did not significantly lower PEP rates compared to controls, with PEP rates of 8.6% and 0.5%, respectively, according to a study in a public hospital in southern Chile [19]. The effectiveness of diclofenac has also been questioned in recent literature; one trial with 1,000 patients revealed a PEP rate of 2.8% compared to historical controls. Variables like patient characteristics, such as obesity, and the intricacy of the case could have impacted these outcomes. Due to inconsistent data regarding its effectiveness, there is ongoing disagreement over the appropriate use of diclofenac in high-risk populations. Preventive measures are being enhanced by combination therapy, like peri-ERCP crystalloid infusion combined with diclofenac [18].

Considering the significant morbidity and mortality implications of ERCP, more research is required to improve PEP management during the procedure and to improve patient selection criteria. In a cohort of 116 patients, divided into two groups: Group A, which included 67 patients without rectal diclofenac, and Group B, which included 49 patients with diclofenac-the study assessed the efficacy of rectal diclofenac in preventing post-ERC pancreatitis (PEP). The average age (61.9 vs. 58.3 years) and gender distribution (68% vs. 69%) did not change significantly. 8.6% of patients experienced PEP; there was no discernible difference in the two groups (four in Group A and six in Group B; p = 0.196). No fatalities or negative diclofenac reactions were reported. Rectal diclofenac did not significantly lower PEP incidence or severity, according to the study's results [19].

According to the research done by Geraci and others, rectal administration of diclofenac, given 30 to 90 minutes prior to ERCP, is a safe and effective way to lower the incidence and severity of pancreatitis following ERCP. It is also a more effective treatment than oral and intramuscular administration of diclofenac sodium enteric-coated capsules. Meta-analyses supporting the effectiveness of diclofenac in reducing post-ERCP pancreatitis (PEP) show a pooled relative risk of 0.36 (95% CI: 0.22-0.60) following treatment. Research indicates that the prevalence of PEP is 3.47%, with 69%, 23%, and 8% of cases classified as mild, moderate, and severe, respectively. When 100 mg of diclofenac is administered rectally, the incidence of PEP is dramatically reduced; rates in high-risk individuals are 6.4% compared to 15.5% [18]. Among the 10 studies, Indomethacin was used in seven and diclofenac was used in three; the pooled RR for PEP with diclofenac is 0.42 (95% CI: 0.23-0.75; p = 0.003) with no statistical heterogeneity (I² = 0%) [23].

Assessing the Effectiveness of Rectal Indomethacin in Preventing Post-ERCP Pancreatitis

The mechanism of post-ERCP pancreatitis (PEP) is thought to involve injury to the papilla from procedures like instrumentation or sphincterotomy, causing edema or spasm, ductal hypertension, and poor pancreatic drainage. This initiates an inflammatory cascade, activates proteolytic enzymes, and releases cytokines (interleukins 1, 6, and 8), leading to systemic inflammation and potential multiorgan involvement [17]. The study explores the efficacy of rectal indomethacin (RI) in preventing post-ERCP pancreatitis (PEP) in both consecutive and low-risk patients. Despite the overall low prevalence of PEP in patients with malignant obstruction, RI showed a significant reduction in PEP occurrence, which contradicted data from another study. The mechanism of PEP is papillary damage, which causes pancreatic duct blockage and subsequent inflammation. RI prevents PEP by suppressing the early steps of the inflammatory cascade [17]. The trial offers strong evidence for Indomethacin's effectiveness in avoiding post-ERCP pancreatitis (PEP). It finds an even more significant 62% reduction in PEP incidence in low-risk individuals, on top of a substantial 42% overall reduction. Additionally, all patient rates of moderate or severe PEP were lowered by 55%, and in low-risk persons, the rates were reduced by 57%. Multivariate analysis demonstrates the effectiveness of Indomethacin despite a greater prevalence of risk variables in the Indomethacin group. Notably, the study's huge sample size lends robustness to its conclusions. In a previous large-scale randomized trial, we compared the efficacy of 100 mg of rectal indomethacin to a placebo in preventing post-ERCP among 602 high-risk patients. This study used the same eligibility criteria and a similar protocol as our current study. The trial showed a 9.2% incidence rate of post-ERCP pancreatitis in the indomethacin group (27 out of 295 patients). For our current study, we estimated that enrolling 1036 patients (518 per group) would give us at least 80% power to detect a 50% reduction in the incidence of post-ERCP pancreatitis, from 9.2% in the 100 mg group to 4.6% in the 200 mg group. This estimation was based on Fisher’s exact test with a two-sided significance level of 0.05 [20].

These findings support earlier studies and highlight the value of Indomethacin as a preventative strategy during ERCP operations. It is imperative to recognize the study's limits, such as its retrospective design and any confounding variables [17]. Despite biostatistical investigations, the effectiveness of rectal Indomethacin in avoiding post-ERCP pancreatitis (PEP) is still debatable [23]. Indomethacin does not undergo significant metabolism during its first pass through the liver, in contrast to diclofenac, which undergoes first-pass metabolism with just 50-60% of the medication reaching systemic circulation intact. Peak serum concentrations obtained within 30-90 minutes and sustained for up to two hours, followed by a steady drop over four hours, lead to complete bioavailability following rectal administration. With the hypothesis that a larger initial dose might result in higher therapeutic drug levels and a more sustained impact, a trial protocol incorporating a high-dose treatment regimen of indomethacin (150 mg immediately post-ERCP followed by an additional 50 mg dose four hours later) was developed. However, it remains uncertain whether administering the entire 200 mg dose immediately post-ERCP, particularly at the beginning of the cascade of events in pancreatitis, might have a more beneficial effect [20].

Some meta-analyses found it unsuccessful, whereas others showed that it was helpful, especially for patients with moderate risk. Significant efficacy was found in both fixed- and random-effects models, according to a subgroup analysis that included one extra RCT. These results highlight the importance of Indomethacin in clinical decision-making by suggesting that it may help prevent PEP. Nonetheless, additional randomized controlled trials and thorough biostatistical analyses are needed to confirm these findings and clarify Indomethacin's effectiveness in PEP prevention. Among the 10 studies, Indomethacin was used in seven and diclofenac was used in three; the pooled RR for PEP with Indomethacin is 0.67 (95% CI: 0.49-0.94; p = 0.02) with low heterogeneity (I² = 30%) [23]. To prevent post-ERCP pancreatitis in high-risk patients, the study evaluated the effectiveness of a high-dose (200 mg) regimen of rectal Indomethacin against the conventional regimen of 100 mg. The incidences of pancreatitis in the two groups were 14.85 in the standard dose group (76/515) and 12.5% in the high-dose group (65/522), with no discernible difference in either group's severity or frequency [20].

Countering Post ERCP Pancreatitis With Rectal NSAIDs

The incidence of PEP was considerably lower in the NSAID group than in the control group. Relative risk (RR): 0.62; 95% confidence interval (CI): 0.46-0.83; p = 0.001. An adjusted indirect treatment comparison revealed no significant difference in efficacy between indomethacin and diclofenac. The risk ratio (RR) was 1.607 (95% CI: 0.824-3.136), demonstrating no significant difference between the two drugs. The findings suggest the use of rectal NSAIDs (indomethacin and diclofenac) for PEP prevention in average-risk individuals with ERCP [23]. The efficacy and toxicity of NSAIDs are dose-dependent, and the best NSAID dose for avoiding PEP is uncertain. A recent randomized, double-blind comparative efficacy trial found that raising the amount of rectal indomethacin to 200 mg was not more effective than the conventional 100 mg dose in decreasing pancreatitis following ERCP in high-risk individuals. Furthermore, there was no discernible distinction in the severity of PEP between the two dosing groups [23].

In a comprehensive analysis of 10 trials involving average-risk patients for post-ERCP pancreatitis (PEP), rectal administration of NSAIDs demonstrated a significant reduction in PEP risk compared to controls (RR = 0.62; 95% CI: 0.46-0.83; p = 0.001), with indomethacin used in seven trials and diclofenac in three. Both indomethacin (RR = 0.67; 95% CI: 0.49-0.94; p = 0.02) and diclofenac (RR = 0.42; 95% CI: 0.23-0.75; p = 0.003) individually exhibited efficacy in PEP prevention [23]. The research study by Bimal Chandra Shil and others emphasizes the value of ERCP in the treatment of biliary and pancreatic disorders, mentioning severe pancreatitis as a frequent side effect. Vital data is presented, showing that 9.78% of people had PEP overall, with a greater frequency in women. It demonstrates the effectiveness of NSAIDs given rectally in conjunction with diclofenac or Indomethacin in lowering the incidence of PEP when compared to no medication intervention.

An ARR of 0.15 (15%), an RRR of 0.75 (75%), and an NNT of 6.5 patients are required to prevent one episode of pancreatitis. This difference is statistically significant (p = 0.014). This result is consistent with several studies and meta-analyses that question the traditional use of pancreatic stents and advocate for additional studies to provide definitive advice. In summary, rectal NSAIDs should be administered immediately after ERCP to prevent PEP in high-risk patients. This highlights the need for more comprehensive trials to advise clinical practice properly [21]. Despite the first contradictory results, the research addresses the substantial influence of periprocedural NSAIDs during ERCP in recent years [22].

Leading worldwide associations encourage routine use of these drugs, especially Indomethacin or diclofenac, with recommendations varying according to patient risk assessment. A substantial decrease in the risk of post-ERCP pancreatitis (PEP), particularly in mild cases, is supported by statistical evidence derived from the pooled results of 21 randomized controlled trials (RCTs). Diclofenac showed an observed risk difference (RD) of -0.05 (95% CI, -0.09 to -0.02; p < 0.05) and an associated number needed to treat (NNT) of 20, while Indomethacin showed an RD of -0.06 (95% CI, -0.10 to -0.02; p < 0.05) and an associated NNT of 17. Rectal administration was the most common method of medication administration. Across 15 studies, 4988 patients favored rectal administration. Overall, 170 out of 2492 patients in the NSAID group and 324 out of 2496 in the control group were provided with PEP. The RD was -0.07 (95% CI: -0.10 to -0.04; P < 0.05). The NTT was 20. The six studies that used other administration routes (PO, IV, and IM) have reported that 80 of 935 patients and 83 of 931 patients in the NSAID and control groups, respectively, presented with PEP. The RD was −0.00 (95% CI, −0.02 to 0.02; p > 0.05). The report highlights the usefulness and cost-efficiency of NSAIDs in reducing PEP overall. Still, it also emphasizes the need for continued research to improve patient outcomes after ERCP and maximize preventive measures. Furthermore, statistical analysis supports the effectiveness of NSAIDs by showing a decrease in PEP incidence when diclofenac, Indomethacin, and other medications are used [22]. 

Limitations

Our search was limited to the English language. It lacks a comprehensive discussion of the potential biases in the data sources used for the research. There was substantial heterogeneity among the studies included in the analysis, mostly concerning the diagnosis and assessment of how PEP could affect outcomes. There were a limited number of RCTs, which may introduce bias and limit the generalizability of findings.

## Conclusions

Our systematic review shows how the effectiveness of rectal NSAIDs, particularly diclofenac and Indomethacin, in preventing post-ERCP pancreatitis remains a topic of debate with conflicting evidence. If given before ERCP, Indomethacin shows promise in lowering PEP rates, whereas the effectiveness of diclofenac is unclear and varies throughout trials. Combination therapy, such as peri-ERCP crystalloid infusion paired with NSAIDs, is being investigated for improved preventive measures. The use of Indomethacin is becoming more and more supported, even in high-risk patients, despite the continuous dispute. The data indicate that these NSAIDs are particularly helpful in preventing mild occurrences of pancreatitis. However, the study stressed the value of conducting additional RCTs to investigate various NSAID administration methods and dosages in order to maximize PEP prevention. However, further research studies are needed to refine patient selection criteria and optimize preventive strategies for the prevention of pancreatitis post-ERCP procedure.

## References

[1] Yadav D, Lowenfels AB (2013). The epidemiology of pancreatitis and pancreatic cancer. Gastroenterology.

[2] Peery AF, Crockett SD, Murphy CC (2019). Burden and cost of gastrointestinal, liver, and pancreatic diseases in the United States: update 2018. Gastroenterology.

[3] Freeman ML, DiSario JA, Nelson DB (2001). Risk factors for post-ERCP pancreatitis: a prospective, multicenter study. Gastrointest Endosc.

[4] Elmunzer BJ, Scheiman JM, Lehman GA (2012). A randomized trial of rectal indomethacin to prevent post-ERCP pancreatitis. N Engl J Med.

[5] Cotton PB, Lehman G, Vennes J (1991). Endoscopic sphincterotomy complications and their management: an attempt at consensus. Gastrointestinal Endoscopy.

[6] Dumonceau JM, Andriulli A, Elmunzer BJ (2014). Prophylaxis of post-ERCP pancreatitis: European Society of Gastrointestinal Endoscopy (ESGE) Guideline - updated June 2014. Endoscopy.

[7] Andriulli A, Leandro G, Niro G (2000). Pharmacologic treatment can prevent pancreatic injury after ERCP: a meta-analysis. Gastrointestinal Endoscopy.

[8] Levenick JM, Gordon SR, Fadden LL (2016). Rectal Indomethacin does not prevent post-ERCP Pancreatitis in consecutive patients. Gastroenterology.

[9] Testoni PA, Mariani A, Aabakken L (2016). Papillary cannulation and sphincterotomy techniques at ERCP: European Society of Gastrointestinal Endoscopy (ESGE) Clinical Guideline. Endoscopy.

[10] Inamdar S, Han D, Passi M, Sejpal DV, Trindade AJ (2017). Rectal indomethacin is protective against post-ERCP pancreatitis in high-risk patients but not average-risk patients: a systematic review and meta-analysis. Gastrointest Endosc.

[11] Liao WC, Angsuwatcharakon P, Isayama H (2017). International consensus recommendations for difficult biliary access. Gastrointest Endosc.

[12] Dumonceau JM, Rigaux J, Kahaleh M, Gomez CM, Vandermeeren A, Devière J (2010). Prophylaxis of post-ERCP pancreatitis: a practice survey. Gastrointest Endosc.

[13] Page MJ, Moher D, Bossuyt PM (2021). PRISMA 2020 explanation and elaboration: updated guidance and exemplars for reporting systematic reviews. BMJ.

[14] Shea BJ, Reeves BC, Wells G (2017). AMSTAR 2: a critical appraisal tool for systematic reviews that include randomised or non-randomised studies of healthcare interventions, or both. BMJ.

[15] Higgins JP, Altman DG, Gøtzsche PC (2011). The Cochrane Collaboration's tool for assessing risk of bias in randomised trials. BMJ.

[16] Gierisch JM, Beadles C, Shapiro A (2014). Health Disparities in Quality Indicators of Healthcare Among Adults with Mental Illness.

[17] Abdelfatah MM, Gochanour E, Koutlas NJ, Hamed A, Harvin G, Othman MO (2020). Rectal indomethacin reduces the risk of post-endoscopic retrograde cholangiopancreatography pancreatitis in low-risk patients. Ann Gastroenterol.

[18] Geraci G, Palumbo VD, D'Orazio B, Maffongelli A, Fazzotta S, Lo Monte AI (2019). Rectal Diclofenac administration for prevention of post-Endoscopic Retrograde Cholangio-Pancreatography (ERCP) acute pancreatitis. Randomized prospective study. Clin Ter.

[19] Losada HF, San Martin PI, Troncoso AI, Silva JA (2021). Rectal diclofenac for prevention of post-endoscopic retrograde cholangiography pancreatitis. Ann Palliat Med.

[20] Fogel EL, Lehman GA, Tarnasky P (2020). Rectal indometacin dose escalation for prevention of pancreatitis after endoscopic retrograde cholangiopancreatography in high-risk patients: a double-blind, randomised controlled trial. Lancet Gastroenterol Hepatol.

[21] Shil BC, Rashid MMU, Saha SK (2021). Efficacy of per rectal non-steroidal anti-inflammatory drugs to prevent post endoscopic retrograde cholangiopancrea-tography pancreatitis: a comparative study. Bang Jr Med.

[22] Serrano JP, de Moura DT, Bernardo WM (2019). Nonsteroidal anti-inflammatory drugs versus placebo for post-endoscopic retrograde cholangiopancreatography pancreatitis: a systematic review and meta-analysis. Endosc Int Open.

[23] Yu S, Shen X, Li L, Bi X, Chen P, Wu W (2021). Rectal indomethacin and diclofenac are equally efficient in preventing pancreatitis following endoscopic retrograde cholangiopancreatography in average-risk patients. JGH Open.

