# Correction: Efficacy of Diclofenac and Indomethacin for Prevention of Post-Endoscopic Cholangiopancreatography (ERCP) Pancreatitis: A Systematic Review

**DOI:** 10.7759/cureus.c358

**Published:** 2025-10-13

**Authors:** Rudrani Kotha, Sabaa I Saad-Omer, Shivani Singh, Oluwatoba T Olayinka, Jaslin Orelus, Mah Rukh Nisar, Sondos T Nassar

**Affiliations:** 1 Internal Medicine, California Institute of Behavioral Neurosciences & Psychology, Fairfield, USA; 2 Neurology/Medicine, California Institute of Behavioral Neurosciences & Psychology, Fairfield, USA; 3 Medicine and Surgery, Jordan University of Science and Technology, Amman, JOR

This article has been corrected to fix the following typo in the PRISMA diagram: '1056 studies identified from registers' was erroneously included in the PRISMA diagram. This has been removed and the PRISMA diagram now accurately reflects the search process as detailed in the article text.

